# Antenatal betamethasone impairs markers of cardiac development and function in near‐term lambs

**DOI:** 10.1113/EP093025

**Published:** 2025-09-04

**Authors:** Reza Amanollahi, Ashley S. Meakin, Stacey L. Holman, Vicki L. Clifton, Kent L. Thornburg, Michael D. Wiese, Kathryn L. Gatford, Mitchell C. Lock, Janna L. Morrison

**Affiliations:** ^1^ Early Origins of Adult Health Research Group, Health and Biomedical Innovation; UniSA: Clinical and Health Sciences University of South Australia Adelaide SA Australia; ^2^ Pregnancy and Development Group, Mater Research Institute University of Queensland South Brisbane Brisbane QLD Australia; ^3^ Department of Medicine, Center for Developmental Health, Knight Cardiovascular Institute, Bob and Charlee Moore Institute of Nutrition and Wellness Oregon Health & Science University Portland Oregon USA; ^4^ Centre for Pharmaceutical Innovation, Clinical & Health Sciences University of South Australia Adelaide SA Australia; ^5^ Robinson Research Institute University of Adelaide Adelaide SA Australia; ^6^ School of Biomedicine University of Adelaide Adelaide SA Australia

**Keywords:** antenatal corticosteroid, cardiac hormones, cardiac metabolism, fetal development, glucocorticoid, sheep

## Abstract

Antenatal corticosteroids are commonly administered to promote fetal lung maturation; however, their impact on heart development is not well understood. This study therefore investigated the effects of antenatal betamethasone on cardiac development in near‐term lambs, using tissues collected from a cohort of ewes with mild experimentally induced asthma. Pregnant ewes received two doses of either saline (Saline) or betamethasone (Betamethasone, intramuscular, 11.4 mg) given 24 h apart, before delivery at 140 days of gestation (term = 150days of gestation). Cardiac protein expression and hormone concentrations in the left ventricle were analysed using western blot and LC–MS/MS, respectively. Fetal and neonatal heart rate and blood pressure were higher in Betamethasone compared to Saline lambs. Betamethasone lambs had lower cardiac concentrations of cortisol, corticosterone, oestradiol, progesterone and thyroxine but a higher triiodothyronine. The protein ratio of glucocorticoid receptor GRβ:GRα‐A was higher in the hearts of Betamethasone compared to Saline. Additionally, the expression of insulin‐like growth factor 1 receptor (a marker of cardiac proliferative capacity), proliferating cell nuclear antigen (a marker of DNA replication) and the fatty acid transporter CD36 were lower in Betamethasone lambs. These findings suggest that antenatal betamethasone may disrupt normal heart development by altering glucocorticoid receptor isoform expression and reducing cardiac exposure to glucocorticoid and sex hormones. Consequently, this leads to decreased expression of markers associated with cardiac growth and fatty acid uptake. These alterations in heart development caused by antenatal corticosteroids exposure may increase the risk of cardiovascular disease later in life if these changes persist into adulthood.

## Abstract (highlights)

- **What is the central question of this study?**
  Does antenatal betamethasone impact heart development?
- **What is the main finding and its importance?**
  Antenatal betamethasone increased blood pressure and disrupted cardiometabolic signals in newborn lambs, suggesting impacts on heart development.

## Graphical abstract

**Abstract figure** Summary of key molecular differences in the left ventricle of near‐term lambs exposed to antenatal betamethasone, compared to saline. Pregnant ewes received two doses of either saline (Saline) or betamethasone (Betamethasone, intramuscular, 11.4 mg) given 24 h apart, before delivery at 140 days of gestation (dG, term = 150 dG). Fetal and neonatal heart rate and blood pressure were higher in Betamethasone compared to Saline lambs. Betamethasone lambs had lower concentrations of cortisol, corticosterone, oestradiol and progesterone in the heart. The protein ratio of glucocorticoid receptor GRβ:GRα‐A was higher in Betamethasone compared to Saline hearts. The expression of IGF1R (a marker of cardiac proliferative capacity) and PCNA (a marker of DNA replication) were also lower, along with the fatty acid transporter CD36 in Betamethasone lambs. These findings highlight that antenatal betamethasone exposure may interfere with normal cardiac maturation, potentially increasing the risk of cardiovascular diseases later in life if these changes persist into adulthood.

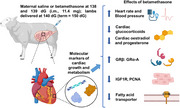

## 1 INTRODUCTION

Antenatal corticosteroids (ACS) are used clinically to reduce the risk of mortality and complications of lung immaturity such as respiratory distress syndrome (RDS) in preterm infants (Mwansa‐Kambafwile et al., 2010; Parker & Dalziel, 2020). International clinical guidelines recommend a single course of ACS treatment (24 mg betamethasone or dexamethasone), given as multiple doses over 24 or 48 h, when preterm birth is anticipated within a week (Antenatal Corticosteroid Clinical Practice Guidelines Panel, 2015; Reddy et al., 2021; WHO, 2022). Betamethasone and dexamethasone are potent synthetic glucocorticoids that remain in their active forms and rapidly cross the placenta into the fetal compartment, as they are poor substrates for the deactivating enzyme 11β‐dehydrogenase type 2 (Diederich et al., 2002; Fee et al., 2023). Effects of ACS on the fetal lung are similar to those of the endogenous glucocorticoid, cortisol. Glucocorticoids induce maturation of the fetal lung by increasing the density of type II alveolar epithelial cells and surfactant production, thinning the alveolar walls and inducing expression of proteins required for lung liquid clearance (Polglase et al., 2007; Wallace et al., 1995). Despite these benefits of ACS for the preterm lung, recent studies show that ACS can induce adverse effects on other organs, such as the developing heart (Jellyman et al., 2020; Sacco et al., 2023). This has functional consequences, as demonstrated in a cohort of 14‐year‐old children who had been born preterm, where those who were exposed to ACS had higher systolic and diastolic blood pressures compared to those who were not exposed (Doyle et al., 2000). This indicates that ACS can change blood pressure not only through potential cardiac effects but also through changing vascular sensitivity to vasoconstrictors. Preclinical studies have also indicated that ACS has adverse impacts on the cardiovascular system, including reduced cardiac output (Fletcher et al., 2002; Miller et al., 2009) and decreased cardiomyocyte proliferation in the heart (Teulings et al., 2020).

Heart development is influenced by hormonal changes during fetal development. In humans and sheep, fetal plasma cortisol concentrations increase near term as part of the natural process of preparing the fetus for life outside the uterus (Fowden et al., 1998; Jellyman et al., 2020). This surge in fetal cortisol functions as a molecular switch for maturation, promoting cell differentiation while reducing further proliferation (Fowden et al., 2016; Liggins, 2000). To initiate signalling, cortisol interacts with the glucocorticoid receptor (GR) to alter the expression of enzymes, transporters and cell signalling molecules (Fowden et al., 2006; Lu et al., 2006). Multiple GR isoforms have been identified in human and sheep placenta and lungs (Clifton et al., 2019; Orgeig et al., 2015; Saif et al., 2014). More recently, we have identified five distinct isoforms in preterm fetal sheep hearts (Amanollahi, Holman, Bertossa, Meakin, Thornburg et al., 2025); however, the associated signalling pathways within the heart and their response to ACS remain largely unexplored.

The molecular mechanisms underlying ACS‐induced cardiometabolic maturation are not well understood. Cardiac metabolism undergoes changes during late gestation heart maturation, with a shift from glycolysis to oxidative phosphorylation (OXPHOS) of fatty acids, resulting in more efficient energy production (Lopaschuk & Jaswal, 2010). The transition from glucose to fatty acids as the primary fuel source is a complex process involving mitochondrial maturation, shifts in substrate availability, altered glucose transporter (GLUT) expression (specifically a reduction in GLUT‐1 (SLC2A1) and an increase in GLUT‐4 (SLC2A4)) and the upregulation of molecules important for fatty acid uptake, such as cluster of differentiation 36 (CD36) and carnitine palmitoyl transferase 1 (CPT1) (Amanollahi, Holman, Bertossa, Meakin, Thornburg et al., 2025; Dimasi et al., 2023; Shao & Tian, 2015). Maternal dexamethasone administration during late gestation increased a protein marker of glycolysis (α‐enolase) in fetal and neonatal rat hearts (Tsuzuki et al., 2009). Chronic maternal cortisol infusion during late gestation reduced mitochondrial abundance in fetal sheep hearts at term, providing further evidence that ACS impacts cardiac energy metabolism before birth (Richards et al., 2014). These findings emphasise the potential for ACS exposure to impact cardiac metabolism and development. Therefore, we hypothesised that antenatal betamethasone in sheep may alter key physiological and molecular markers of cardiac function, growth and metabolism, interfering with normal heart development.

## 2 METHODS

### 2.1 Ethics and animal husbandry

This study was approved by the South Australian Health and Medical Research Institute (SAHMRI) Animal Ethics Committee (SAM455.19) and was conducted in accordance with the Australian Code of Practice for the Care and Use of Animals for Scientific Purposes (National Health & Medical Research Council, 2013), the ARRIVE guidelines (Percie du Sert et al., 2020), the ethical principles outlined by Grundy (2015) and the principles of 3Rs (Tannenbaum & Bennett, 2015). Merino ewes were kept in paddocks with natural grazing throughout the experiment, and managed as previously described (Robinson et al., 2024; Roff et al., 2025). Ewes were time mated to Merino rams.

### 2.2 Prenatal treatments

Ewes in the present cohort were all from our previously reported cohort where mild maternal asthma was induced (Hammond et al., 2025; Roff et al., 2025). To match mothers for antenatal exposure, in the present study we compared outcomes in lambs from asthmatic ewes that had received either saline or betamethasone antenatally. Betamethasone treatment was evaluated in these near‐term pregnancies as a therapeutic approach to induce fetal lung maturation (Robinson et al., 2024), given that we had observed indices of lung immaturity including lower surfactant protein expression in near‐term sheep fetuses from mothers with experimental asthma (Clifton et al., 2016; Wooldridge et al., 2019). Nevertheless, in the present cohort, where the maternal asthma phenotype was much milder, lamb lung function and maturation were similar in offspring of asthmatic and non‐asthmatic control ewes (Robinson et al., 2024). The ewes were randomised via a random number generator to receive either saline or betamethasone (Celestone Chronodose 11.4 mg, Schering Plough, Baulkham Hills, NSW, Australia) by intramuscular injections 48 and 24 h prior to delivery, consistent with current clinical practice guidelines (Antenatal Corticosteroid Clinical Practice Guidelines Panel, 2015). To prevent preterm labour caused by betamethasone‐induced progesterone withdrawal, all ewes received a 150 mg intramuscular injection with medroxyprogesterone acetate at 6–9 days prior to antenatal injections (Jenkin et al., 1985; Jobe et al., 2003; Robinson et al., 2024). Lambs were delivered via Caesarean section at 140 ± 2 days of gestation (dG; term = 150 dG) and ventilated for 45 min using a volume guarantee strategy, as previously described (Robinson et al., 2024). Arterial and venous catheters were connected to LabChart 7 (ADInstruments, Bella Vista, NSW, Australia) for continuous blood pressure monitoring as previously described (Robinson et al., 2024). Heart rate (HR) and blood pressure (BP; systolic blood pressure, SBP; diastolic blood pressure, DBP; and mean arterial pressure, MAP) were measured in the fetuses and neonates in 4–5 min blocks. In this study we analysed fetal HR and BP data (during the 4 min prior to delivery) as well as in neonates (at 30–34 min post‐delivery), the period during stable ventilation when artifact‐free data were available for the maximum number of animals. A total of 26 lambs from singleton (*n* = 8) and twin (*n* = 9) pregnancies were included in the study.

### 2.3 Post‐mortem and tissue collection

At the end of the ventilation study, the lambs were humanly killed with an overdose of sodium pentobarbitone (20 mg kg^−1^, Virbac Australia, Peakhurst, NSW, Australia), the heart was removed and left ventricle (LV), right ventricle (RV) and septum were dissected and weighed. The tissues were then either flash frozen in liquid nitrogen for molecular studies or fixed in 4% paraformaldehyde for histological studies. In this study, we used LV tissues from the lambs whose mothers were treated with either saline (Saline, *n* = 14; eight males (M), six females (F)) or betamethasone (Betamethasone, *n* = 12; 5M, 7F).

### 2.4 Quantification of hormone concentrations in the neonatal heart

Tissue hormone concentrations were determined by liquid chromatography (LC; Shimadzu Nexera XR, Shimadzu, Japan) coupled to a SCIEX 6500 Triple‐Quad system (MS/MS; SCIEX, US) using an adapted protocol (Dimasi, Darby, Cho et al., 2024; McBride et al., 2021). Neonatal LV tissue was homogenised in 500 µL 0.9% NaCl at 50 Hz for 2 min and then centrifuged at 12,000 *g* for 10 min at 4°C. Supernatant (100 µL) was added to 300 µL acetonitrile containing 50 ng/mL internal standard (cortisol‐9,11,12,12‐d4; Toronto Research Chemicals, Toronto, Canada), vortexed for 1 min and then centrifuged at 12,000 *g* for 10 min. The supernatant was transferred to a fresh Eppendorf tube and the remaining pellet was resuspended in 300 µL ethyl acetate. After vortexing for 1 min, the sample was centrifuged at 12,000 *g* for 10 min. The supernatant was combined with the acetonitrile, mixed by inversion, and evaporated to dryness using the GeneVac EZ‐2 Evaporating System (GeneVac, Ipswich, UK). Dried samples were reconstituted in 50% methanol and then injected onto an ACQUITY UPLC BEH C18 Column (130 Å, 1.7 µm, 2.1 mm × 100 mm; Waters, Milford, MA, USA). Mobile phases were 0.1% formic acid in water (A) and 0.1% formic acid in acetonitrile (B). Flow rate was 0.3 mL/min, initially at 10% mobile phase B, which was increased linearly to 90% over 10 min and then held at 90% for 2 min, after which it returned to 10% for 3 min prior to injection of the next sample. Hormone concentrations were determined by integrating with a standard curve ranging from 0.05 to 100 ng/mL. The detection conditions for analytes were consistent with previously described methods (Dimasi, Darby, Holman et al., 2024; Lock et al., 2023; McBride et al., 2021; Meakin et al., 2024; Robinson et al., 2024).

### 2.5 Quantification of protein expression in the neonatal heart

Approximately 100 mg of neonatal LV tissue was cut and homogenised via sonication (John Morris Scientific, Sydney, NSW, Australia) in a lysis buffer containing Tris–HCl (50 mM), NaCl (150 mM), NP‐40 (1%), sodium orthovanadate (1 mM), sodium fluoride (30 mM), sodium pyrophosphate (10 mM), EDTA (10 mM), and protease inhibitor (one tablet/20 mL buffer; complete Mini, Roche, Basel, Switzerland). The homogenised tissues were centrifuged at 14,300 *g* for 14 min at 4°C using an Eppendorf Centrifuge 5415 (Crown Scientific, Melbourne, VIC, Australia), and the supernatant was transferred into new microtubes. The protein concentration of each sample was determined via a Pierce Micro BCA Protein Assay Kit (Thermo Fisher Scientific Inc., Waltham, MA, USA), using bovine serum albumin for the standard curve. To quality check for protein loading, diluted samples (5 mg mL^−1^) were resolved using a 12% SDS‐PAGE gel and stained with Coomassie blue. To probe target proteins, equal concentration (75 µg) of each sample were resolved via SDS‐PAGE (10–15%) and transferred onto a nitrocellulose membrane (Hybond ECL, GE Healthcare, Mascot, NSW, Australia), followed by a wash with Tris‐buffered saline (TBS) for 5 min. To visualise the transferred total proteins, the membranes were stained with Ponceau S solution (0.1% (w/v) in 5% acetic acid, Sigma‐Aldrich, St. Louis, MA, USA) and imaged using the ImageQuant LAS4000 system (GE Healthcare, Melbourne, VIC, Australia). The membranes were washed with TBS (3 × 5 min), then blocked in 5% BSA in TBS with 1% Tween (TBS‐T) for 1 h at room temperature. After another wash with TBS‐T (3 × 5 min), membranes were cut according to the size of the protein of interest and incubated with one of the following primary antibodies overnight at 4°C. We used primary antibodies that target proteins of interest including proteins that determine cardiac mitochondrial activity: total OXPHOS (1:500, cat. no. ab110413, Abcam, Cambridge, UK), MitoBiogenesis cocktail (1:250, cat. no. ab123545, Abcam), peroxisome proliferator‐activated receptor γ coactivator 1‐α (PGC‐1α, 1:1000, cat. no. 2178S, Cell Signaling Technology, Danvers, MA, USA), manganese superoxide dismutase (Mn‐SOD, 1:1000, cat. no. 06984, Merk); glucocorticoid signalling: total GR (1:1000, cat. no. A303‐491A, Bethyl Laboratories, Montgomery, TX, USA), GRβ (1:500, cat. no. PA3‐514, Invitrogen), 11β‐hydroxysteroid dehydrogenase type 1 (11βHSD‐1, 1:1000, cat. no. ab36364, Abcam), 11βHSD‐2 (1:1000, cat. no. 10004549, Cayman Chemical Co., Ann Arbor, MI, USA); glucose metabolism: insulin receptor substrate 1 (IRS‐1, 1:1000, cat. no. 3194, Cell Signaling Technology), p‐IRS‐1(Ser789) (1:1000, cat. no. 2389S, Cell Signaling Technology), Akt substrate of 160 (AS160, 1:1000, cat. no. 2670S, Cell Signaling Technology), p‐AS160(Thr642) (1:1000, cat. no. 4288S, Cell Signaling Technology), GLUT‐1 (1:2000, cat. no. NB300‐666SS, Novus Biologicals, Centennial, CO, USA), GLUT‐4 (1:1000, cat. no. ab33780, Abcam); fatty acid metabolism: pyruvate dehydrogenase kinase 4 (PDK‐4, 1:1000, cat. no. PA5‐79800, Thermo Fisher Scientific), CD36 (1: 1000, cat. no. ab133625, Abcam), CPT1 (1: 1000, sc‐98834, Santa Cruz Biotechnology, Dallas, TX, USA); cardiac contractility: phospholamban (PLN, 1:1000, cat. no. 14562S, Cell Signaling Technology), p‐PLN(Ser16/Thr17) (1:1000, cat. no. 8496S, Cell Signaling Technology), sarco/endoplasmic reticulum‐type calcium transport ATPase 2 (SERCA2, 1:1000, cat. no. ab137020, Abcam), troponin‐I (1:1000, cat. no. 4002, Cell Signaling Technology), and p‐troponin‐I(Ser23/24) (1:1000, cat. no. 4004S, Cell Signaling Technology); and cardiac growth: insulin‐like growth factor 1 receptor (IGF1R, 1:1000, cat. no. 3027S, Cell Signaling Technology), proliferating cell nuclear antigen (PCNA, 1:2000, cat. no. 2586, Cell Signaling Technology), cyclin D1 (1:1000, cat. no. 55506S, Cell Signaling Technology), ribosomal protein S6 kinase (P70 S6K, 1:1000, cat. no. 9202, Cell Signaling Technology), p‐P70 S6K(Thr389) (1:1000, cat. no. 9205, Cell Signaling Technology), Forkhead box protein O1 (FOXO1, 1:1000, cat. no. 9454, Cell Signaling Technology), and p‐FOXO1(Thr24) (1:1000, cat. no. 9464, Cell Signaling Technology), as previously described (Amanollahi, Holman, Bertossa, Meakin, Thornburg et al., 2025; Botting et al., 2014; Darby et al., 2022; Lie et al., 2014; Orgeig et al., 2015; Padhee et al., 2023; Saif et al., 2014; Wang et al., 2015; Zhang et al., 2024). All antibodies were diluted in 5% BSA in TBS‐T at the concentrations recommended by the manufacturer. The blots were then washed with TBS‐T (3 × 5 min) before being incubated with the appropriate horseradish peroxidase (HRP)‐conjugated secondary IgG antibody for 1 h at room temperature. Bands were visualised using enhanced chemiluminescent (ECL) HRP substrate (SuperSignal West Pico, Thermo Fisher Scientific) and imaging with ImageQuant LAS4000 (GE Healthcare). The protein abundance was quantified by densitometry using ImageQuant TL 8.1 software (GE Healthcare). The reference protein on each membrane was either Vinculin (stained at 1:2000, cat. no. 18799S, Cell Signaling Technology) or β‐tubulin (stained at 1:1000, cat. no. 5346, Cell Signaling Technology) depending on target protein molecular mass. For each blot, housekeeping proteins were selected based on the molecular mass of the target proteins. The expression of the housekeeping proteins was also assessed between the groups to ensure consistent expression. In cases where the housekeeping protein was unreliable due to either variations or interference from the protein of interest, we normalised the target proteins to total protein using Ponceau S staining.

### 2.6 Quantification of glycogen and collagen staining in the neonatal heart

Paraformaldehyde‐fixed paraffin‐embedded blocks of neonatal LV tissue were sectioned at 5 µm onto charged slides, baked at 60°C for 1 h, followed by deparaffinisation and rehydration, and stained with periodic acid‐Schiff (PAS) or Masson's trichrome. Stained slides were scanned at ×40 magnification using a NanoZoomer‐XR (Hamamatsu 2.0‐HT, Hamamatsu, Japan) to create digitised whole‐slide images. The glycogen‐positive area was analysed in images of PAS‐stained tissue using the Fiji/ImageJ software suite (version 1.54f, NIH, Bethesda, MD, USA). Five frames were randomly selected from each slide at ×20 magnification, ensuring they were at least 1 mm apart. The ‘Colour Threshold’ tool was utilised to assess the glycogen area as a percentage of the total tissue area, with results averaged over the five frames per animal (Darby et al., 2018; Lock et al., 2019). The collagen‐positive area (%) was determined on images of Masson's trichrome‐stained slides using custom colour thresholds in Visiopharm software (Visiopharm version 2020.08.4.9377, Hoersholm, Denmark). The analysis was conducted by a researcher who was blinded to the treatment groups.

### 2.7 Quantification of Ki67 staining in the neonatal heart

Neonatal LV tissue was sectioned at 5 µm onto charged slides. Slides were baked at 60°C for 1 h, followed by deparaffinisation and rehydration. After rehydration, endogenous peroxide activity was inhibited by pre‐treating slides with 3% hydrogen peroxide in phosphate‐buffered saline for ∼20 min (Sigma‐Aldrich), before performing heat‐induced antigen retrieval (∼20 min at 121°C; 2100Retriever, Aptum Biologics, Southampton, UK) in citrate buffer (pH 6.0). The slides were then incubated overnight at 4°C with the primary antibody (Ki67, 1:200, cat. no. M7240, Agilent Technologies, Santa Clara, CA, USA) after blocking non‐specific binding with a blocking solution (eBioscience cat. no. 00‐4953‐54, Thermo Fisher Scientific). Negative control slides, which omitted the primary antibody, were included to confirm the absence of non‐specific binding of the secondary antibody and reagent contamination. Additional negative controls involved substituting the primary antibody with mouse serum (Sigma‐Aldrich). Positive cells were visualised using a Metal Enhanced DAB Substrate Kit (cat. no. 34065, Thermo Fisher Scientific), and all sections were counterstained with Mayer's haematoxylin (Sigma‐Aldrich). The stained slides were scanned using a NanoZoomer‐XR (Hamamatsu) and digital images were analysed with QuPath software (version 0.4.4) to quantify the percentage of Ki67 positive cells (Lock et al., 2019, 2015; Morrison et al., 2007). The analysis was conducted by a researcher who was blinded to the treatment groups.

### 2.8 Statistical analyses

Outliers were detected using Grubbs’ test (α = 0.05) and omitted prior to subsequent analyses using SPSS Statistics v29 (IBM Corp., Armonk, Armonk, NY, USA). Data was analysed by a linear mixed model using treatment and lamb sex as factors; ewe was included as a random factor to correct for the effects of the common maternal environment in twins. *Post hoc* mixed models were used to determine effects of treatment within each sex. To assess the correlation between two measures, simple linear regression was used. Some samples were not included in some analyses due to missing animal records (fetal parameters), systematic or technical errors (hormone assay). In the western blot data, any non‐quantifiable bands (due to an artifact on the blots) were excluded from the analysis; these are indicated with a X on images of blots below. Data are presented as means (SD). A *P*‐value of <0.05 was considered statistically significant.

## 3 RESULTS

### 3.1 Maternal betamethasone increased fetal and neonatal heart rate and blood pressure

**FIGURE 1:**
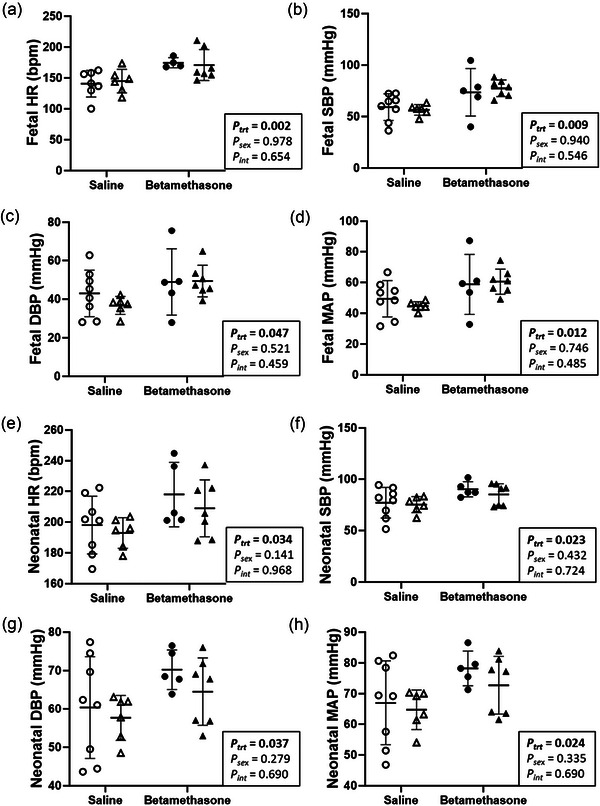
Maternal betamethasone increased fetal and neonatal heart rate and blood pressure. Males (M), circles; females (F), triangles. Ewes were treated with either saline (open symbols; *n* = 8M, 6F) or betamethasone (filled symbols; *n* = 5M, 7F) at 48 and 24 h before delivery of progeny. Heart rate (HR; bpm, beats per minutes) and blood pressure (SBP, systolic blood pressure; DBP, diastolic blood pressure; MAP, mean arterial pressure) were measured during the final 4 min before delivery in fetuses and from 30 to 34 min post‐delivery in neonates. Up to one outlier was excluded per group using Grubbs's method (α = 0.05), when applicable. Data were analysed by a linear mixed model using treatment and lamb sex as factors; ewe was included as a random factor to correct for the effects of the maternal environment. *P *< 0.05 was considered significant. Bars and whiskers indicate means (SD).

Heart rate (HR) and blood pressure (BP) were measured to evaluate cardiac autonomic and haemodynamic function. Prior to caesarean delivery, fetal HR (*P *= 0.002), SBP (*P *= 0.009), DBP (*P *= 0.047) and MAP (*P *= 0.012) were higher in betamethasone than saline fetuses (Figure 1A–D). Neonatal HR (*P *= 0.034), SBP (*P *= 0.023), DBP (*P *= 0.037) and MAP (*P *= 0.024) were also higher in Betamethasone compared to Saline lambs (Figure 1E–H). Measures of fetal and neonatal heart rate and blood pressure did not differ between males and females (*P* > 0.1 for all, Figure 1).

### 3.2 Maternal betamethasone affected neonatal heart growth

Heart and ventricle weights were measured to assess cardiac hypertrophy and structural remodelling. Absolute LV weight was lower (*P *= 0.027), while RV weight relative to body weight (BW) was higher in Betamethasone than Saline lambs (*P *= 0.006, Table 1). Although there was a significant interaction between treatment and sex (*P* = 0.045), LV:BW ratio (Table 1) did not differ between treatments in either males (*P* = 0.097) or females (*P* = 0.720). Lamb body weight and other measures of heart weight and proportions were not affected by treatment or sex (*P* > 0.1 for all, Table 1).

**TABLE 1:** Maternal betamethasone affected neonatal heart growth.

	Saline		Betamethasone		Significance		
	Male (*n* = 8)	Female (*n* = 6)	Male (*n* = 5)	Female (*n* = 7)	*P*_trt_	*P*_sex_	*P*_int_
BW (kg)	4.73 (0.69)	4.54 (0.54)	4.52 (0.79)	4.12 (0.74)	0.386	0.474	0.980
Heart (g)	31.4 (6.5)	28.27 (3.73)	29.91 (8.05)	27.62 (4.70) (*n* = 6)	0.721	0.364	0.768
Heart: BW	6.59 (0.65)	6.25 (0.82)	6.58 (1.02)	7.06 (0.56) (*n* = 6)	0.223	0.761	0.184
LV (g)	11.87 (1.52) (n 7)	11.09 (1.21)	9.80 (1.35)	10.30 (1.71)	**0.027**	0.817	0.298
LV: Heart	0.38 (0.05) (n 7)	0.39 (0.05)	0.34 (0.10)	0.37 (0.01) (*n* = 6)	0.362	0.652	0.780
LV: BW	2.47 (0.22) (n 7)	2.45 (0.32)	2.23 (0.53)	2.53 (0.41)	0.440	0.993	0.045^*^
RV (g)	8.48 (1.69)	7.86 (1.11)	9.03 (1.42)	8.19 (1.02)	0.454	0.226	0.841
RV: Heart	0.27 (0.02)	0.28 (0.05)	0.30 (0.03)	0.29 (0.02) (*n* = 6)	0.102	0.877	0.427
RV: BW	1.78 (0.14)	1.73 (0.21)	2.01 (0.22)	2.01 (0.25)	**0.006**	0.768	0.784
Septum (g)	4.51 (1.61)	3.75 (0.67)	3.57 (1.09)	3.69 (1.05)	0.309	0.520	0.370
Septum: Heart	0.14 (0.05)	0.13 (0.04)	0.11 (0.01)	0.12 (0.01) (*n* = 6)	0.221	0.923	0.631
Septum: BW	0.96 (0.36)	0.83 (0.17)	0.77 (0.12)	0.89 (0.16)	0.526	0.927	0.232

Data are expressed as means (SD) and were analysed by a linear mixed model using treatment and lamb sex as factors; ewe was a random factor to correct for the effects of the maternal environment. Number of animals is shown in brackets where data was not available from all lambs due to missing records. *P < *0.05 was considered statistically significant (indicated in bold). ^*^LV: BW did not differ between treatments in either males (*P* = 0.097) or females (*P* = 0.720). Abbreviations: BW, body weight; LV, left ventricle; RV, right ventricle.

### 3.3 Maternal betamethasone dysregulated concentrations of hormones in the neonatal left ventricle

**FIGURE 2:**
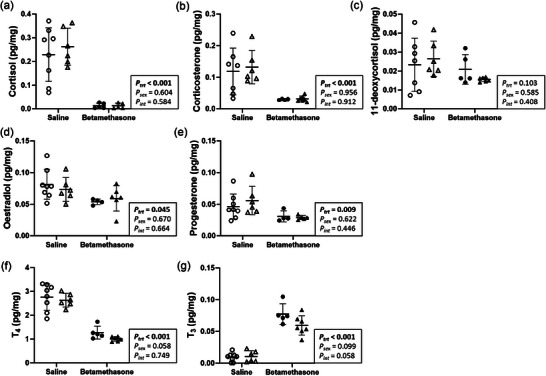
Maternal betamethasone dysregulated concentrations of hormones in the neonatal left ventricle. Males (M), circles; females (F), triangles. Ewes were treated with either saline (open symbols; *n* = 8M, 6F) or betamethasone (filled symbols; *n* = 5M, 7F) at 48 and 24 h before delivery of progeny. Up to one outlier was excluded per group using Grubbs's method (α = 0.05), when applicable. Data were analysed by a linear mixed model using treatment and lamb sex as factors; ewe was included as a random factor to correct for the effects of the maternal environment. *P *< 0.05 was considered significant. Bars and whiskers indicate means (SD). T_4_, thyroxine. T_3_, triiodothyronine.

Cardiac concentrations of hormones including glucocorticoids, sex and thyroid hormones were measured to evaluate endocrine regulation of cardiac development. Left ventricle concentrations of cortisol (*P *< 0.001, Figure 2a) and corticosterone (*P *< 0.001, Figure 2b), but not 11‐deoxycortisol (*P* = 0.103, Figure 2c), were lower in Betamethasone than Saline lambs. Concentrations of oestradiol (*P *= 0.045, Figure 2d), progesterone (*P *= 0.009, Figure 2e), and thyroxine (T_4_, *P *< 0.001, Figure 2f) were lower, and triiodothyronine (T_3_, *P *< 0.001, Figure 2g) were higher in Betamethasone compared to Saline lambs. Hormone concentrations in the left ventricle did not differ between males and females (*P* > 0.05 for all, Figure 2).

### 3.4 Maternal betamethasone dysregulated glucocorticoid receptors in the neonatal left ventricle

**FIGURE 3:**
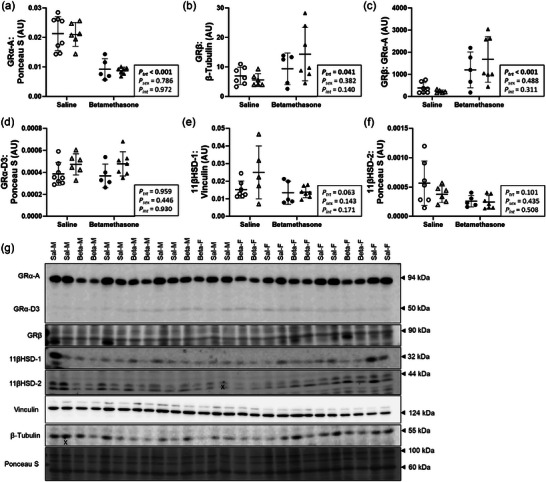
Maternal betamethasone dysregulated glucocorticoid receptors in the neonatal left ventricle. Males (M), circles; females (F), triangles. Ewes were treated with either saline (open symbols; *n* = 8M, 6F) or betamethasone (filled symbols; *n* = 5M, 7F) at 48 and 24 h before delivery of progeny. Up to one outlier was excluded per group using Grubbs's method (α = 0.05), when applicable. Data were analysed by a linear mixed model using treatment and lamb sex as factors; ewe was included as a random factor to correct for the effects of the maternal environment. *P *< 0.05 was considered significant. Bars and whiskers indicate means (SD). AU: arbitrary unit. ‘X’ indicates data excluded from analysis (due to an artifact on the band/s). 11βHSD, 11β‐Hydroxysteroid dehydrogenase; GR, glucocorticoid receptor.

Cardiac protein expression of GR isoforms was measured to assess glucocorticoid signalling in cardiac regulation and adaptation. Left ventricle protein expression of GRα‐A was lower (*P *< 0.001, Figure 3a), while GRβ was higher (*P *= 0.041, Figure 3b) in Betamethasone compared to Saline lambs, and not different between males and females. The ratio of GRβ: GRα‐A (*P* < 0.001, Figure 3c) was higher in Betamethasone compared to Saline lambs, regardless of sex. Protein expression of GRα‐D3, 11βHSD‐1 and 11βHSD‐2 did not differ between treatments or sexes (*P* > 0.05 for all, Figure 3d–f). Figure 3g shows western blot images of target and reference proteins.

### 3.5 Maternal betamethasone downregulated markers of cardiac growth in the neonatal left ventricle

**FIGURE 4:**
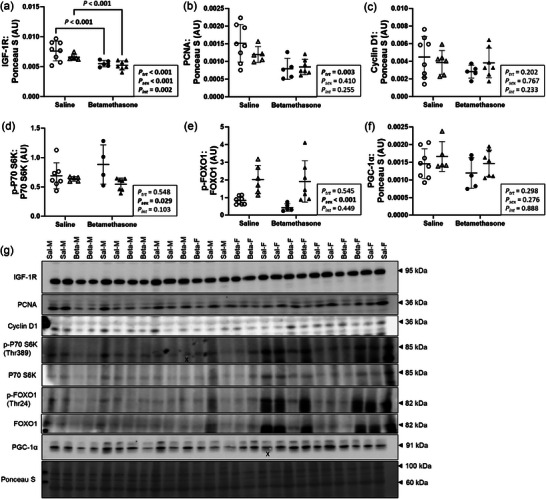
Maternal betamethasone downregulated markers of cardiac growth in the neonatal left ventricle. Males (M), circles; females (F), triangles. Ewes were treated with either saline (open symbols; *n* = 8M, 6F) or betamethasone (filled symbols; *n* = 5M, 7F) at 48 and 24 h before delivery of progeny. Up to one outlier was excluded per group using Grubbs's method (α = 0.05), when applicable. Data were analysed by a linear mixed model using treatment and lamb sex as factors; ewe was included as a random factor to correct for the effects of the maternal environment. *P *< 0.05 was considered significant. Bars and whiskers indicate means (SD). AU: arbitrary unit. ‘X’ indicates data excluded from analysis (due to an artifact on the band/s). FOXO1, forkhead box protein O1; IGF1R, insulin‐like growth factor 1 receptor; p‐, phosphorylated; P70S6K, ribosomal protein S6 kinase; PCNA, proliferating cell nuclear antigen; PGC‐1α, peroxisome proliferator‐activated receptor γ coactivator 1‐α.

Cardiac markers of proliferation and hypertrophy were measured to evaluate cellular growth and structural remodelling. Effects of treatment on left ventricle protein expression of IGF1R differed between sexes (*P* = 0.002, Figure 4a), and expression of IGF1R was lower in Betamethasone compared to Saline lambs within each sex (each *P *< 0.001). Protein expression of PCNA (*P *= 0.003, Figure 4b) was lower in Betamethasone compared to Saline lambs, and did not differ between males and females (*P *= 0.410). Protein expression of cyclin D1 (Figure 4c) did not differ between treatments (*P *= 0.202) or sexes (*P *= 0.767). P70 S6K phosphorylation (Figure 2d) and FOXO1 (Figure 2e) did not differ between treatments (*P* = 0.548 and *P* = 0.545, respectively). P70 S6K phosphorylation was higher in males than females (*P* = 0.029), whilst FOXO1 phosphorylation was higher in females than males (*P* < 0.001). Protein expression of PGC‐1α (Figure 4f) did not differ between treatments (*P* = 0.298) or sexes (*P* = 0.276). Figure 4g shows western blot images of target and reference proteins.

### 3.6 Maternal betamethasone dysregulated markers of glucose and fatty acid uptake in the neonatal left ventricle

**FIGURE 5:**
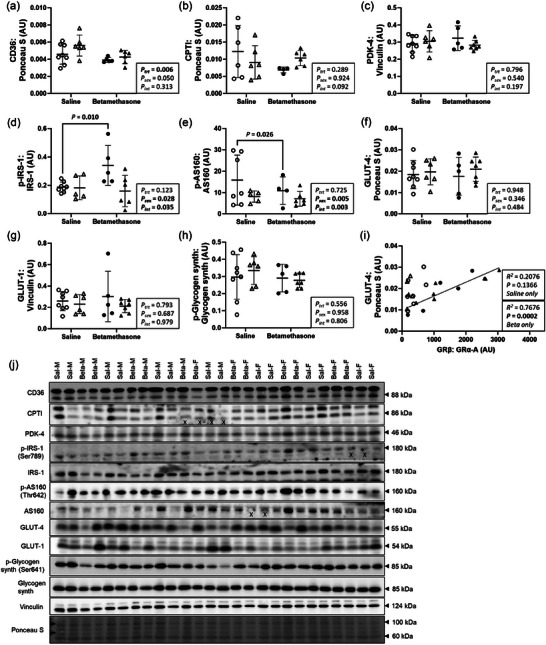
Maternal betamethasone dysregulated markers of glucose and fatty acid uptake in the neonatal left ventricle. Males (M), circles; females (F), triangles. Ewes were treated with either saline (open symbols; *n* = 8M, 6F) or betamethasone (filled symbols; *n* = 5M, 7F) at 48 and 24 h before delivery of progeny. Up to one outlier was excluded per group using Grubbs's method (α = 0.05), when applicable. Data were analysed by a linear mixed model using treatment and lamb sex as factors; ewe was included as a random factor to correct for the effects of the maternal environment. To assess the correlation between two measures, simple linear regression was used. *P *< 0.05 was considered significant. Bars and whiskers indicate means (SD). AU: arbitrary unit. ‘X’ indicates data excluded from analysis (due to an artifact on the band/s). AS160, Akt substrate of 160 kDa; CD36, cluster of differentiation 36; CPT1, carnitine palmitoyl transferase 1; GLUT, glucose transporter; IRS‐1, insulin receptor substrate 1; p‐, phosphorylated; PDK‐4, pyruvate dehydrogenase kinase 4.

Cardiac markers of glucose and fatty acid uptake were measured to assess metabolic function and substrate utilisation. Left ventricle protein expression of CD36 (*P *= 0.006, Figure 5a) was lower in Betamethasone compared to Saline lambs, and did not differ between males and females (*P* = 0.050). Protein expression of CPT1 (Figure 5b) and PDK‐4 (Figure 5c) did not differ between treatments (*P* = 0.289 and *P* = 0.796, respectively) or sexes (*P* = 0.924 and *P* = 0.540 respectively). Effects of treatment on IRS‐1 phosphorylation differed between sexes (*P* = 0.035, Figure 5d), and expression of IRS‐1 phosphorylation was higher in Betamethasone compared to Saline males (*P *= 0.010), and did not differ between treatments within females (*P* = 0.685). Effects of treatment on AS160 phosphorylation differed between sexes (*P* = 0.003, Figure 5e) and expression of AS160 phosphorylation was lower in Betamethasone compared to Saline males (*P *= 0.026), and did not differ between treatments within females (*P* = 0.095). Protein expression of GLUT‐4 (Figure 5f), GLUT‐1 (Figure 5g), and glycogen synthase phosphorylation (Figure 5h) did not differ between treatments or sexes (*P* > 0.1 for all). There was a positive correlation between GRβ:GRα‐A and GLUT‐4 in Betamethasone lambs only (*R* ^2^ = 0.7676, *P* = 0.0002, Figure 5i). Figure 5j shows western blot images of target and reference proteins.

### 3.7 Males exhibited higher protein expression of OXPHOS complexes I–IV in the neonatal left ventricle than females, regardless of treatment

**FIGURE 6:**
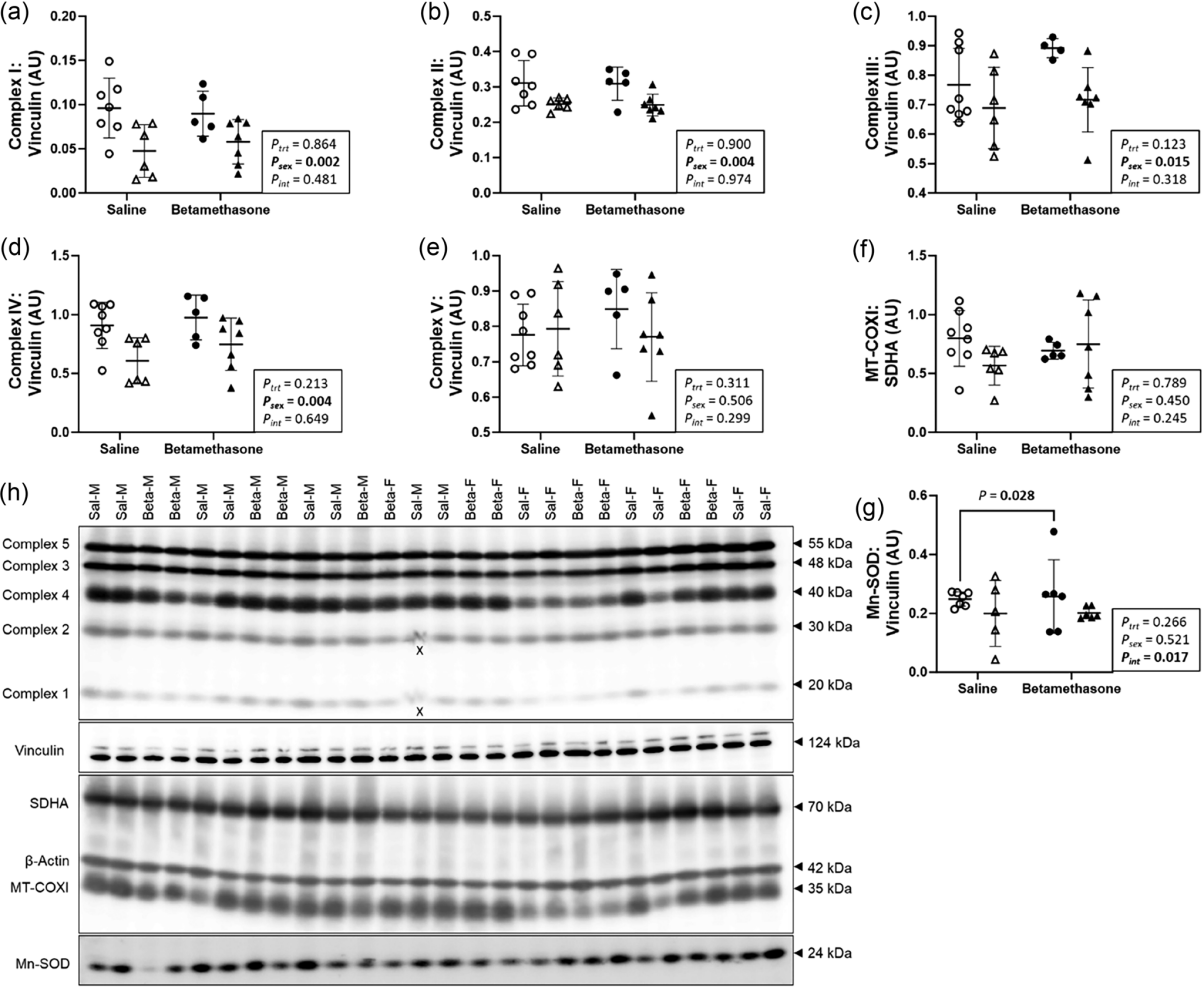
Males exhibited higher protein expression of OXPHOS complexes I–IV in the neonatal left ventricle than females, regardless of treatment. Males (M), circles; females (F), triangles. Ewes were treated with either saline (open symbols; *n* = 8M, 6F) or betamethasone (filled symbols; *n* = 5M, 7F) at 48 and 24 h before delivery of progeny. Up to one outlier was excluded per group using the Grubbs's method (α = 0.05), when applicable. Data were analysed by a linear mixed model using treatment and lamb sex as factors; ewe was included as a random factor to correct for the effects of the maternal environment. *P *< 0.05 was considered significant. Bars and whiskers indicate means (SD). AU: arbitrary unit. ‘X’ indicates data excluded from analysis (due to an artifact on the band/s). MT‐COXI:SDHA protein ratio, a marker of mitochondrial abundance; Mn‐SOD, manganese superoxide dismutase.

Cardiac markers of OXPHOS and mitochondrial content were measured to evaluate mitochondrial function and oxidative energy metabolism. Left ventricle protein expression of OXPHOS complexes I (*P *= 0.002, Figure 6a), II (*P *= 0.004; Figure 6b), III (*P *= 0.015, Figure 6c), and IV (*P *= 0.004, Figure 6d), but not complex V (*P *= 0.506, Figure 6e), was higher in males compared to females, and did not differ between treatments (*P* > 0.1 for all). Protein expression of MT‐COXI:SDHA was not affected by treatment (*P* = 0.789) or sex (*P* = 0.450, Figure 6f). Effects of treatment on protein expression of Mn‐SOD differed between sexes (*P *= 0.017, Figure 6g), and expression of Mn‐SOD was higher in Betamethasone compared to Saline males (*P *= 0.028), and did not differ between treatments within females (*P* = 0.780). Figure 6h shows western blot images of target and reference proteins.

### 3.8 Maternal betamethasone did not alter markers of contractile function in the neonatal left ventricle

**FIGURE 7:**
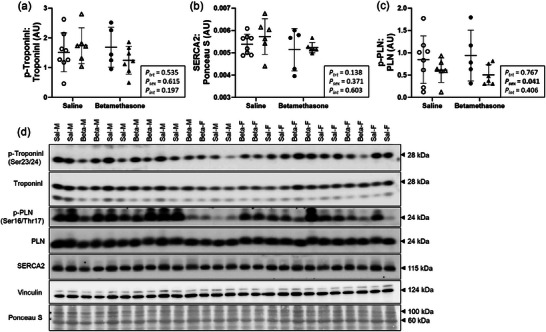
Maternal betamethasone did not alter markers of contractile function in the neonatal left ventricle. Males (M), circles; females (F), triangles. Ewes were treated with either saline (open symbols; *n* = 8M, 6F) or betamethasone (filled symbols; *n* = 5M, 7F) at 48 and 24 h before delivery of progeny. Up to one outlier was excluded per group using the Grubbs's method (α = 0.05), when applicable. Data were analysed by a linear mixed model using treatment and lamb sex as factors; ewe was included as a random factor to correct for the effects of the maternal environment. *P *< 0.05 was considered significant. Bars and whiskers indicate means (SD). AU: arbitrary unit; p‐, phosphorylated; PLN, phospholamban; SERCA2, sarco(endo)plasmic reticulum calcium‐ATPase 2.

Cardiac markers of contractility were measured to assess the functional capacity of cardiac contraction. Left ventricle protein expression of Troponin‐I phosphorylation (Figure 7a) and SERCA2 (Figure 7b) did not differ between treatments (*P* = 0.535 and *P* = 0.138, respectively) or sexes (*P* = 0.615 and *P* = 0.371, respectively). Protein expression of PLN phosphorylation was higher in males compared to females (*P* = 0.041, Figure 7c), and did not differ between treatments (*P* = 0.767). Figure 7d shows western blot images of target and reference proteins.

### 3.9 Maternal betamethasone did not alter proportions of glycogen, collagen or Ki67 staining in the neonatal left ventricle

**FIGURE 8:**
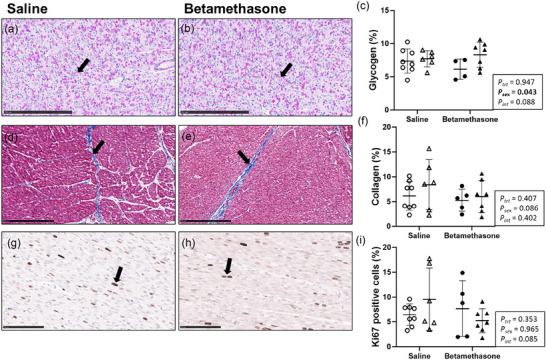
Maternal betamethasone did not alter proportions of glycogen, collagen or Ki67 staining in the neonatal left ventricle. Males (M), circles; females (F), triangles. Ewes were treated with either saline (open symbols; *n* = 8M, 6F) or betamethasone (filled symbols; *n* = 5M, 7F) at 48 and 24 h before delivery of progeny. ×20 magnification representative micrograph of glycogen staining by PAS (a, b, scale bars, 250 µm). ×20 magnification representative micrograph of collagen staining by Masson's trichrome (d, e, scale bars 250 µm). ×40 magnification representative micrograph of Ki67 staining by IHC (g, h, scale bars, 100 µm). Up to one outlier was excluded per group using Grubbs's method (α = 0.05), when applicable. Data were analysed by a linear mixed model using treatment and lamb sex as factors; ewe was included as a random factor to correct for the effects of the maternal environment. *P *< 0.05 was considered significant. Bars and whiskers indicate means (SD).

Cardiac staining of glycogen, collagen and Ki67 was measured to evaluate metabolic storage, fibrotic remodelling and cellular proliferation. Left ventricle proportion of glycogen (*P* = 0.043, Figure 8a–c) staining was higher in females compared to males but did not differ between treatments (*P* = 0.947). The proportions of collagen (Figure 8d–f) and Ki67 (Figure 8g–i) staining did not differ between treatments (*P* = 0.407 and *P* = 353, respectively) or sexes (*P* = 0.086 and *P* = 0.965, respectively).

## 4 DISCUSSION

Antenatal betamethasone exposure was associated with higher fetal and neonatal heart rate and blood pressure, lower concentrations of endogenous glucocorticoid and sex hormones as well as increased protein ratio of GRβ:GRα‐A in the neonatal heart. Additionally, the cardiac growth markers IGF1R and PCNA, along with the fatty acid transporter CD36, were downregulated in LV tissue from betamethasone‐exposed lambs, suggesting impaired growth and energy metabolism. These findings indicate that while antenatal betamethasone supports lung development, it may disrupt cardiac maturation, potentially predisposing offspring to long‐term cardiovascular risks.

In the present study, administration of a clinical course of maternal betamethasone before near‐term birth increased fetal and neonatal heart rate and blood pressure. This aligns with findings in baboons where exposure of the developing fetus to exogenous betamethasone at 0.7 of gestation elevated fetal blood pressure within 6 h of the first dose and blood pressure remained higher throughout the 72 h study period (Koenen et al., 2002). Moreover, 2 days of high dose cortisol infusion, similar in duration to our betamethasone exposure, induced severe fetal hypertension and increased hypertrophic growth of the fetal heart in sheep (Giraud et al., 2006; Louey & Thornburg, 2005). Our observed increase in the RV:BW in betamethasone‐exposed lambs may be attributed to elevated blood pressure, potentially leading to right‐sided hypertrophic growth in the heart. Our observation of RV but not LV hypertrophy may reflect the greater sensitivity of the RV to afterload than the LV (Karamlou et al., 2019).

Alterations in heart development may also be mediated by changes in cardiac hormone concentrations. We also observed that maternal betamethasone decreased concentrations of multiple cardiac hormones in near‐term lambs including cortisol and corticosterone. This is similar to the reduction in endogenous glucocorticoids in neonatal plasma and lung tissue that we have reported in the same cohort (Robinson et al., 2024). Most studies investigating endocrine impacts of ACS have reported fetal/maternal plasma concentrations of hormones, with limited data available on cardiac tissue, highlighting a gap in understanding the role of steroid hormones, including cortisol, in heart development. Consistent with suppression of fetal cortisol production after ACS treatment in sheep in the present study, maternal administration of betamethasone in pregnant women reduced cortisol concentrations in maternal plasma, cord plasma and amniotic fluid within 24 h after injection (Ballard et al., 1975; Jeffray et al., 1999; Marinoni et al., 1998). Since glucocorticoids bind to tissue GR and exert negative feedback on the hypothalamic–pituitary–adrenal (HPA) axis (Almanza‐Sepulveda et al., 2020; Lu & Cidlowski, 2006), it is likely that ACS suppresses endogenous glucocorticoid production through the same mechanism.

This study revealed that the cardiac concentrations of oestradiol (the most potent oestrogen) and progesterone were lower in newborn lambs exposed to betamethasone. Oestrogen and progesterone are essential for fetal heart development, ensuring its ability to adapt to the physiological demands of life after birth. Oestrogen regulates critical processes such as cardiac protection (Dubey & Jackson, 2001; Patten & Karas, 2006), angiogenesis (Morales et al., 1995) and cardiomyocyte proliferation (Kim et al., 2006). Progesterone inhibits cardiomyocyte apoptosis (Morrissy et al., 2010), promotes proliferation and cardiac repair (Lan et al., 2020), and modulates the contractile units of the heart (Moshal et al., 2014). Taken together, lower concentrations of oestradiol and progesterone during fetal heart development may impair cardiac maturation and alter cardiovascular function, which may in turn increase susceptibility to postnatal cardiovascular diseases. The observed reduction in cardiac oestradiol and progesterone concentrations in betamethasone‐exposed lambs could be related to the suppression of the neonatal hypothalamic–pituitary–gonadal (HPG) axis, potentially as an indirect consequence of HPA axis suppression (Kapoor et al., 2008). Supporting this, a study in guinea pigs demonstrated that maternal glucocorticoid treatment programmes HPA axis regulation in adult offspring, along with alterations in gonadal function, highlighting significant interactions between synthetic glucocorticoid and the developing HPG axis (Liu et al., 2001). Other studies showed that prenatal administration of betamethasone leads to postnatal changes in the ovine HPA axis that persist into adulthood (Sloboda et al., 2002), and that cortisol infusion suppressed oestradiol production in ewes (Breen et al., 2005). Collectively, these previous studies support our findings that betamethasone has the potential to reduce sex hormone concentrations.

We also found lower cardiac T_4_ alongside higher cardiac active T_3_ concentrations in lambs exposed to antenatal betamethasone. Consistent with our findings, maternal dexamethasone administration at clinically relevant doses increased plasma T_3_ concentrations without affecting T_4_ concentrations in fetal sheep (Forhead et al., 2007). The authors concluded that the elevated circulating T_3_ may result from both enhanced hepatic conversion of T_4_ to T_3_ and decreased clearance of T_3_ by the kidney and placenta (Forhead et al., 2007). During late fetal life, increasing circulating cortisol drives increases in deiodinase activity in sheep, resulting in decreasing fetal plasma T_4_ concentration as it is converted to the more active form T_3_ (Chattergoon et al., 2012; Forhead et al., 2006). Interestingly, increased T_4_ to T_3_ ratios in the present cohort of lambs were observed despite lower cardiac cortisol concentrations, suggesting that betamethasone mimics cortisol's role in stimulating cardiac deiodinase activity.

To our knowledge, our study is the first to describe multiple GR isoforms in the neonatal sheep heart. We observed lower cardiac protein expression of GRα‐A, higher GRβ and similar GRα‐D3 in lambs exposed to betamethasone, compared to lambs whose mothers received saline. GRα‐A is the primary functional isoform that regulates a variety of genes and proteins through its interaction with glucocorticoid response element (Lu & Cidlowski, 2005). We have recently identified five distinct GR isoforms (GRα‐A, GR‐P, GR‐A, GRα‐D2 and GRα‐D3) in the fetal sheep heart, with their protein abundance decreasing as gestational age advanced (Amanollahi, Holman, Bertossa, Meakin, Thornburg et al., 2025). Moreover, in a sheep model of preterm birth, ACS downregulated expression of the NR3C1 gene (encoding the GR) in the heart (Ivy et al., 2021), which is consistent with our observation of lower GRα‐A protein in betamethasone‐exposed lambs. Higher protein expression of GRβ in betamethasone‐exposed lambs may have a similar effect, since this isoform does not bind glucocorticoid agonists nor activate glucocorticoid‐responsive reporter genes in the nucleus (Kino et al., 2009). When co‐expressed with GRα, GRβ functions as a dominant negative regulator, antagonising the activity of GRα on many glucocorticoid‐responsive genes (Oakley & Cidlowski, 2013). The observed combination of lower GRα‐A and higher GRβ expression in the fetal heart after exposure to betamethasone may therefore reduce glucocorticoid sensitivity in the neonatal heart. While this altered balance of GR isoforms may represent a protective mechanism against glucocorticoid overexposure in ACS‐exposed fetuses, it could also impair critical glucocorticoid‐mediated processes essential for cardiac maturation.

Antenatal betamethasone also lowered protein expression of IGF1R (a marker of cardiac proliferative capacity) and PCNA (a marker of DNA replication) in the present study. Neonatal dexamethasone treatment in rats led to reduced adult heart weight, cardiomyocyte hypertrophy and myocardial fibrosis at 50 weeks of age (de Vries et al., 2002; Kamphuis et al., 2001). The reduction in cardiac mass in these rats was attributed to the suppression of physiological cardiomyocyte proliferation following neonatal glucocorticoid exposure (de Vries et al., 2006). It is worth noting that cardiomyocyte proliferation continues well into the neonatal period in rats, whereas sheep and human cardiomyocytes transition from a proliferative state to terminal differentiation during late gestation (Bergmann et al., 2009; Dimasi et al., 2023; Guo & Pu, 2020; Jonker et al., 2010; Lock et al., 2018). Terminal differentiation of cardiomyocytes forms mature cells that function for life but have limited capacity for regeneration or repair postnatally (Botting et al., 2012; Dimasi et al., 2023; Morrison et al., 2018). Similar to the rat, exposure to ACS can adversely affect the cardiovascular system in preterm human infants, causing transient hypertrophic cardiomyopathy (Werner et al., 1992). These effects were transient, peaking by the third week of treatment and returning to near pretreatment levels by the sixth week (Werner et al., 1992); however, cardiomyocyte proliferation cannot be studied in the human neonate. Since the number of cardiomyocytes is fixed before birth in sheep, as in humans, the reduction in cardiac growth markers observed in our study suggests that betamethasone exposure during the in utero developmental period may limit the heart's ability to adapt and function optimally later in life.

Cardiac protein expression of CD36 (a marker of fatty acid uptake) was downregulated in lambs that were exposed antenatally to betamethasone. During the fetal to postnatal transition, the heart shifts from glucose and lactate to fatty acids as its primary energy source, enabling more efficient ATP production via CD36‐mediated fatty acid β‐oxidation (Lopaschuk & Jaswal, 2010; Palm et al., 2022). Downregulation of CD36 in response to antenatal betamethasone therefore suggests a disruption in the transition from fetal to postnatal energy substrate utilisation, potentially delaying the maturation of cardiac metabolism. Consistent with impacts of ACS on cardiac metabolism, ACS during late pregnancy in mice reduced the gene expression of GR in the fetal heart 24 h post‐exposure and disrupted the normal upregulation of genes related to fatty acid metabolism (Ivy et al., 2021). These authors concluded that ACS may interfere with fetal heart maturation by downregulating the ability to respond to glucocorticoid (Ivy et al., 2021). Another study using the sheep model of early onset of fetal growth restriction (FGR) showed that there is significant downregulation in mRNA expression of fatty acid metabolism markers *CD36* and *FABP* (encode fatty acid binding protein) in the fetal heart (Dimasi, Darby, Cho et al., 2024). Interestingly, we also found a positive correlation between the GRβ:GRα‐A protein ratio and GLUT‐4 in betamethasone‐exposed hearts. This reinforces our speculation that betamethasone reduces glucocorticoid sensitivity in the heart, potentially increasing reliance on glucose uptake over fatty acids and disrupting cardiac metabolism. To support this, maternal dexamethasone was associated with a decrease in endogenous fetal plasma cortisol concentrations, as well as hyperglycaemia and hyperinsulinemia in sheep fetuses at 70% of gestation (106–107 dG, term = 150 dG, Gray et al., 2006). These disruptions in the fetal metabolic and hormonal environment were associated with increased GLUT‐1 and GLUT‐4 protein expression in fetal skeletal muscle (Gray et al., 2006).

Despite ACS‐induced changes in fatty acid metabolism, the influence of ACS on components of the mitochondrial ATP‐generating pathway were not detected. However, regardless of treatment, protein expression of complexes I–IV was higher in males than in females, suggesting that male hearts may have a greater OXPHOS capacity than female hearts. This finding is supported by studies in mice, human and sheep demonstrating that cardiac mitochondrial DNA and protein levels are higher in males compared to females (Cao et al., 2022; Dimasi, Darby, Cho et al., 2024). In the present study, regardless of treatment, males also exhibited higher cardiac PLN phosphorylation, which may enhance SERCA pump activity and facilitate faster relaxation in male hearts compared to female hearts. In line with this, a study on mice also found that protein expression of SERCA2 and PLN phosphorylation were higher in males compared to females, indicating sex difference in cardiac contractile function and intracellular Ca^2+^ homeostasis (Ceylan‐Isik et al., 2011). We also found that in betamethasone‐exposed lambs, IRS‐1 phosphorylation was higher, while AS160 phosphorylation was lower in males compared to females. This suggests that males may exhibit altered insulin signalling and glucose metabolism in the heart in response to in utero betamethasone exposure. Similarly, maternal exposure to dexamethasone or cortisol during early pregnancy has distinct effects on insulin secretion and glucose regulation in adult male sheep offspring, suggesting that males may be more vulnerable than females to prenatal glucocorticoid exposure (De Blasio et al., 2007). In the present study, betamethasone‐exposed males also had higher cardiac protein expression of Mn‐SOD than females, which may indicate an adaptive response to increased oxidative stress in the heart. Evidence suggests that excess glucocorticoids can induce oxidative stress in vascular tissue and elicits vascular endothelial dysfunction (Iuchi et al., 2003).

We previously investigated similar cardiometabolic pathways in a study examining cortisol infusion in fetal sheep during mid–late gestation (109–116 dG), a preterm period (Amanollahi, Holman, Bertossa, Meakin, Clifton et al., 2025). Here, we briefly compare key findings from that study with the current one to highlight how gestational timing and ligand type (synthetic vs. endogenous glucocorticoid) influence cardiac programming. Cortisol infusion increased local cardiac cortisol concentrations (Amanollahi, Holman, Bertossa, Meakin, Clifton et al., 2025), whereas betamethasone significantly reduced endogenous cortisol concentrations. Exogenous glucocorticoid, cortisol in preterm (Amanollahi, Holman, Bertossa, Meakin, Clifton et al., 2025) and betamethasone in near‐term fetal sheep, induced changes in cardiac expression of GR isoforms that would be expected to downregulate GR signalling. Cortisol infusion downregulated IGF1R and SIRT1 protein expression while upregulating PCNA abundance in the preterm fetal heart, suggesting an acceleration of cardiac maturation, likely mediated by altered GR isoform expression (Amanollahi, Holman, Bertossa, Meakin, Clifton et al., 2025). Interestingly, in contrast, antenatal betamethasone decreased protein abundance of both IGF1R and PCNA in near‐term hearts. In previous work, we demonstrated that the prepartum or an earlier rise in cortisol supports cardiac maturation, a process marked by increased cardiomyocyte binucleation and elevated PCNA expression (a marker of DNA replication) (Amanollahi, Holman, Bertossa, Meakin, Clifton et al., 2025; Amanollahi, Holman, Bertossa, Meakin, Thornburg et al., 2025). Thus, the reduction in PCNA expression observed with betamethasone exposure may reflect impaired cardiomyocyte binucleation. Metabolically, GLUT‐4 protein expression was upregulated by cortisol (Amanollahi, Holman, Bertossa, Meakin, Thornburg et al., 2025) but remained unchanged with betamethasone. However, in the betamethasone‐exposed hearts, there was a positive correlation between the GRβ:GRα‐A ratio and GLUT‐4, suggesting that betamethasone reduces glucocorticoid sensitivity, potentially shifting the heart's reliance toward glucose uptake. Additionally, we observed downregulation of CD36 in response to antenatal betamethasone, suggesting a disruption in the normal metabolic transition from fetal to postnatal energy substrate utilisation. Overall, these findings suggest differences in impacts of cortisol and betamethasone on key cardiometabolic pathways. However, further studies are needed to elucidate whether these differences are due to the timing of heart development or reflect differences between cortisol and betamethasone actions.

### 4.1 Limitations and future directions

While this study provides valuable insights into the effects of antenatal betamethasone on cardiac development in neonatal lambs, some limitations should be acknowledged and could be explored in future studies. In this study, we used offspring from asthmatic ewes treated with either saline or betamethasone. Although we have previously shown that mild maternal asthma did not affect fetal lung function or structure (Robinson et al., 2024), the impact of maternal asthma alone on fetal heart development is not known. We suggest that this question warrants investigation in future studies. Another limitation of the study is that we were not able to measure fetal or neonatal cardiac function due to the short duration our neonatal ventilation protocol, which precluded adding magnetic resonance imaging to the study (Cho et al., 2024, 2020; Schrauben et al., 2021).

Cardiac hormones were measured at the end of the study in neonatal tissue, and betamethasone‐induced changes were not assessed longitudinally. Moreover, while this study investigated multiple hormonal and molecular markers, it does not encompass the complete array of factors influencing cardiac growth and function. For example, high‐resolution respirometry of mitochondrial OXPHOS activity and reactive oxygen species production was not performed as the study utilised snap‐frozen tissue from a completed study, and fresh tissue was not available. The physiological data were also obtained under acute/anaesthetised conditions. Molecular markers were examined in the LV due to its central role in postnatal cardiac workload; however, future studies could aim to include the RV to provide understanding of effects of ACS across multiple cardiac regions. Finally, the long‐term consequences of altered cardiac development due to betamethasone exposure were not evaluated, warranting further longitudinal studies to elucidate the lasting impacts on cardiovascular health in adulthood.

## 5 Conclusion

This study reveals that antenatal betamethasone exposure increases heart rate and blood pressure in neonatal sheep, as well as disrupting multiple hormonal and molecular pathways critical for heart development. The suppression of key cardiac hormones including cortisol and oestradiol suggests that betamethasone modulates the HPA and HPG axes, potentially impairing the heart's ability to fully mature and maintain cardioprotection. The downregulation of GRα‐A and cardiac growth markers further highlight the impact of betamethasone on cardiac growth and maturation. Additionally, the downregulation of CD36, a key transporter for fatty acid uptake, suggests that ACS exposure delays maturation of cardiac metabolism, disrupting the transition from fetal glucose dependence to postnatal fatty acid utilisation. These findings highlight the necessity for judicious use of ACS, given their potential to alter the developmental trajectory of the heart, potentially increasing susceptibility to cardiovascular disease later in life.

## AUTHOR CONTRIBUTIONS

Conception or design of the work: Ashley S. Meakin, Vicki L. Clifton, Michael D. Wiese, Kathryn L. Gatford, Mitchell C. Lock, Janna L. Morrison. Acquisition or analysis or interpretation of data for the work: Reza Amanollahi, Ashley S. Meakin, Stacey L. Holman, Vicki L. Clifton, Kent L. Thornburg, Michael D. Wiese, Kathryn L. Gatford, Mitchell C. Lock, Janna L. Morrison. Drafting the work or revising it critically for important intellectual content: Reza Amanollahi, Kathryn L. Gatford, Mitchell C. Lock, Janna L. Morrison. All authors have read and approved the final version of this manuscript and agree to be accountable for all aspects of the work in ensuring that questions related to the accuracy or integrity of any part of the work are appropriately investigated and resolved. All persons designated as authors qualify for authorship, and all those who qualify for authorship are listed.

## CONFLICT OF INTEREST

The authors declare no conflicts of interest.

## Data Availability

All data supporting the results are presented in the manuscript.
